# Consequences of COVID-19 on Employees in Remote Working: Challenges, Risks and Opportunities An Evidence-Based Literature Review

**DOI:** 10.3390/ijerph191811672

**Published:** 2022-09-16

**Authors:** Clara De Vincenzi, Martina Pansini, Bruna Ferrara, Ilaria Buonomo, Paula Benevene

**Affiliations:** Department of Human Studies, Libera Università Maria SS. Assunta, 00193 Rome, Italy

**Keywords:** workplace healthy, long COVID-19, well-being, economy

## Abstract

The COVID-19 pandemic forced organizations across all sectors and sizes to undertake crucial changes in order to remain productive during the emergency. Among these, the shift towards remote working arrangements is still present in our workplaces, impacting employees’ well-being and productivity. This systematic review aims to describe the pandemic’s consequences on work organization by analyzing whether and how the shift towards remote or home-working impacted employees’ productivity, performance, and well-being. Furthermore, it describes the role of individual and organizational factors in determining employees’ adjustment to remote work. Sixty-seven peer-reviewed papers published from 2020 to 2022, written in English, were selected through the preferred reporting items for systematic reviews and meta-analyses (PRISMA) guidelines. Findings describe how remote working arrangements, the workplace and organizational factors, and the employees’ individual traits and skills impacted employees’ productivity and well-being. Furthermore, they provide a description of the organizational enforcement actions reported in the literature. Managerial and practical implications, such as enforcement actions, team management strategies, and initiatives to promote employees’ physical and mental health, will be discussed in the paper.

## 1. Introduction

At the end of 2019, the coronavirus disease 2019 (COVID-19) became a pandemic: it began in Wuhan, China, but rapidly spread all over the world. On 30 January 2020, the World Health Organization (WHO) declared the outbreak of the COVID-19 pandemic as a public health emergency of international concern [1]. Considering the severe consequences of the COVID-19 pandemic on individuals’ physical health as well as on public health and social systems, governments adopted strict prevention measures. There were however differences between countries, also in relation to the different phases of the COVID-19 pandemic [2]. Some people could not go outside of their houses, while in other countries lockdowns did not last long and reached only specific economic sectors [3]. Among the most recurrent measures to contrast the COVID-19 pandemic was the mandatory closure of schools, the interruption of all nonessential productions and commercial activities, and the transformation of the workplace from a physical office space into a virtual place [4].

The national lockdowns that were implemented to stop the spread of the virus forced many organizations to turn suddenly into remote work, pushing towards a much greater use of technology [5]. At the same time, before the pandemic, most workers had little remote working experience, nor were they or their organizations prepared to support these practices [6].

The sudden spread of technology-based working arrangements resulted in a high number of international reports and scientific papers describing employees’ working conditions, using several different terms, such as remote working [7,8], teleworking [9,10], and working from home [11,12]. Despite the differences among them (for example, remote or teleworking not necessarily imply that the employee is working from home), it is not possible to universally define technology-based work arrangements [13,14,15]. For this reason, throughout this paper we will use remote working and teleworking as synonyms and will refer to work from home or home-working when the papers we are citing used such terms.

Apart from the issues linked to definitions of remote working, most organizations were not ready or willing to move in this direction. Nevertheless, the changes endorsed because of the pandemic generated a new workplace panorama. It is improbable that the previous organizational assets will be restored due to the substantial changes that the pandemic has somehow imposed and promoted. Current research and reports, indeed, suggest that remote working or, more generally, flexible forms of work will be implemented even after the pandemic’s end. In other words, different forms of hybrid work are becoming a stable feature of the workplace [16,17].

In this light, it is interesting to understand the challenges, opportunities, and risks related to remote working that impacted organizations during the COVID-19 onset and spread [18]. The organizational changes enforced now are the result of two processes. On one hand, they refer to organizations’ answers to an exceptional period, to find ways to survive or even grow. On the other, they are new forms of working that have proven to be effective in the short run but require attention regarding employees’ well-being and organizational productivity. It is then essential to understand which lessons are to be learned from remote work, which seems to be the most relevant change introduced in the workplace [9].

Of course, the different lockdowns or periods in which a number of restrictions were imposed were themselves a stressful event. However, the push towards remote work and, in general, towards new ways of working linked with information and communication technology (ICT) emerged during this phase and generated knowledge and experiences that need to be capitalized upon [19]. Therefore, this stage is highly needed: the phase of dramatic change may be over, but there are changes due to the widespread remote work that will still continue to have an impact.

Work organization has dramatically been re-arranged, following different schemes, depending on different dimensions: the availability of ICT and employees’ skills before the pandemic; the actual feasibility of this change according to the sector in which the organizations operate. For example, the remote working transition was possible mainly among office-based work but not among production plants or manufacturing sites [5,10]. Consistently, an ILO policy brief published in 2020 showed that only 20 to 34% of the workforce could work from home, based on the tasks and characteristics of different types of jobs [20].

The relative feasibility of the remote transition for office-based organizations implied that some organizations or employees moved from office-based work to full-time home-based work [21,22,23]. In some cases, employees were allowed to choose whether they wanted to work from home; in other instances, it was required either by national laws to counter the pandemic or it was mandatory as a result of organizational decisions [24,25]. Finally, there were different phases, where employees had to stay at home, while in other periods, employees could go back partially or totally to the office to work [26].

While in some cases the tasks involved in the remote working phase were just the same as the ones pursued before the pandemic onset, in most cases, new tasks were added due to the general changes faced by the organizations [27,28]. Furthermore, new strategies were implemented to introduce higher flexibility or innovation, for example, when establishing new forms of social support from colleagues or supervisors to overcome difficulties and problems arising from ICT use [27,28].

In any of these cases, remote or home-based work requires changes in the strategies implemented to monitor employees’ work and performance. Remote working makes it impossible to use well-known, office-based performance evaluation strategies [29,30].

Overall, studies and reports on the pandemic’s impact on organizations (across all sectors and sizes) highlight the profound changes that organizations were forced to undertake to remain productive during the different lockdowns [8,31,32,33]. As stated before, these changes still inform our workplaces. Thus, this review aims to describe the pandemic’s consequences on work organization by analyzing whether and how the shift towards remote or home-working impacted the employees’ productivity, performance, and well-being. Furthermore, even in consideration of the unexpectedness of the COVID-19-related events, this study describes the role of individual and organizational factors in determining employees’ adjustment to remote work.

## 2. Methods

The paper selection process followed the preferred reporting items for systematic reviews and meta-analyses (PRISMA) statement [34]. Eligibility criteria were empirical studies published in peer-reviewed full-length articles from 2020 to 2022, written in English. The period of literary research lasted from May 2020 to July 2022.

### 2.1. Information Sources and Search Strategy

Databases and search engines employed for the search were: EBSCOhost, ProQuest, and Web of Science. Each database required a different detailed strategy. At the same time, the following generic combination of keywords covered the focus of our research:

“Remote working” or “telework” or “eworking” or “e-working” or “work from home” or “home-based tele-work” or “virtual working” or “telecommuting” or “smart working” or “agile working” or “agile work” or “smart work” or “teleworking” or “ework” or “e-work” or “home working” or “home work” or “home-based work” or “home based work” or “home-based working” or “home based working” “home-working” or “home-work”

And

“Psychosocial risks” or “well-being” or “wellbeing” or “stress” or “technostress” or “tecnostress”

The keywords were searched in the publication title or abstract according to the needs.

### 2.2. Data Collection Process

All references were gathered in a Mendeley database. Two authors independently reviewed selected references, thus obtaining the final list of documents to be analyzed. As the chosen databases allowed to preselect full-text availability, year, and language of publication, this manual selection procedure mainly regarded paper content. Papers in which the content was not entirely within the scope of this review (e.g., theoretical position paper, prescriptive approaches, best practices) and did not include empirical research were eliminated. Furthermore, the authors scrutinized the reference section of selected papers, looking for further works written in English that could fit the eligibility criteria. They also read their abstracts to check whether they could be included in the review.

**Figure 1:**
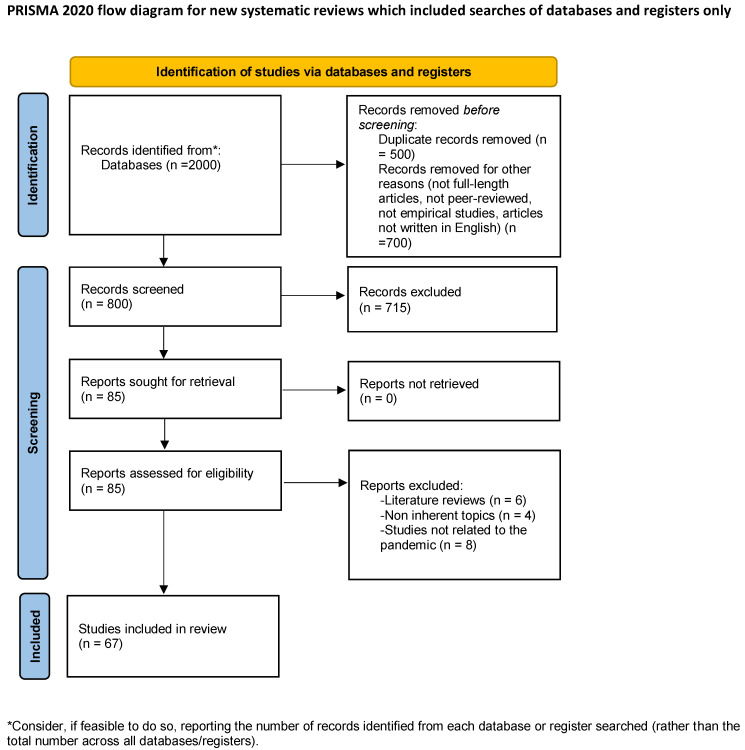
Study selection workflow.

Figure 1 [35] shows the whole workflow that brought about the final paper selection.

### 2.3. Study Selection

After applying the inclusive and exclusive criteria (Figure 1), 67 papers were determined as eligible and were included in the review (included papers are marked with an asterisk in the references list).

## 3. Results

**Figure 2:**
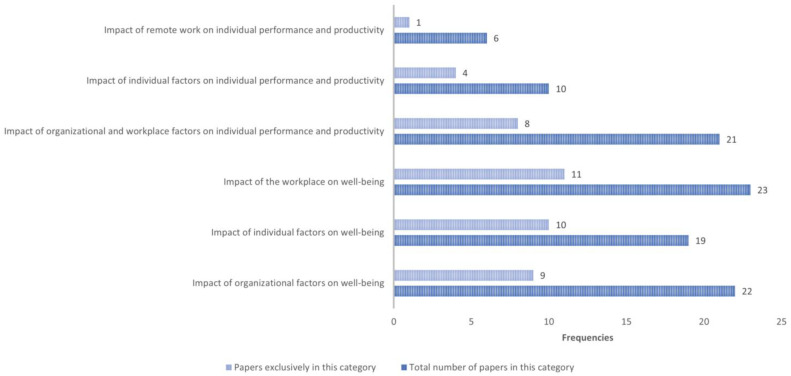
Papers distribution across categories.

Seven main themes emerged from the content analysis of the papers. Figure 2 shows how the papers are distributed across the categories. As shown, almost all the papers (N = 64) refer to employee well-being conditions, whether impacted by the workplace, the individual or the organizational factors. A total of 37 papers, instead, described the impact of remote working experiences and conditions on productivity and performance. Furthermore, Figure 2 indicates how many papers include contents specifically related to one category among the ones individuated (Papers exclusively in this category in Figure 2). With this regard, it is interesting to observe that several papers focus on one category (N of papers addressing only one category = 43), with categories on the impact on employee well-being being the most represented across the papers.

### 3.1. Impact of Remote Work on Individual Performance and Productivity

Several research contributions among the ones selected for this review describe the effects of remote or home-working on employee productivity.

Before considering the effects of such working arrangements on performance and productivity, a first reflection on the performance assessments has to be done. From the paper analysis, it emerges that the assessment by employees of their quality of work and productivity is not the same across different professional groups. For example, in a study in Italy, teachers reported a lower perceived quality of work than other professionals [36]. On the other hand, when the job tasks are usually pursued in an office, the work-from-home arrangement seems to boost the perceived performance [36]. These differences likely depend on the tasks usually performed at work and the ICT literacy of the employees. In the case of teachers, despite having potential good ICT skills because of the use of technology in the classroom, videoconferencing tools to interact with students may have negatively impacted the quality of their work.

Concerning the effects of remote working on employees, many selected papers describe issues related to stress and anxiety conditions, mainly due to the consequences of full or partial national lockdowns that forced remote or home-work, or the fast switch to the use of ICT. Overall, such conditions affected employees’ well-being and, in turn, their productivity [19,37].

At the same time, some studies showed that when employees were satisfied with their telework conditions, they experienced higher subjective well-being and better self-reported performance [38]. Further, subjective well-being emerged also to partially mediate the relationship between telework satisfaction and self-reported performance [38].

Interestingly, in a study performed among a group of employees working from home in Hong Kong, stress did not directly impact productivity but promoted non-work-related activities during working hours, such as caring for children, doing housework, or playing video games and sports. However, performing these non-work-related activities did not affect productivity, suggesting that this may effectively counteract stress [39].

In this regard, the participants of a Czech study reported that they found their work from home more efficient than in the office. Moreover most of the 90 employees interviewed showed a positive perception of home office employees with regards to saving time, or the feeling of freedom, while confirming the adverse effects of isolation [40].

These findings are interesting since they show that remote work per se does not bring positive or negative outcomes, but rather that the consequences depend on many individual and work factors. Therefore, the following sections will describe individual and organizational factors that contribute to shaping the effect of remote working on employee performance during the pandemic.

### 3.2. Impact of Individual Factors on Individual Performance and Productivity

In line with the previous literature on job satisfaction, employees’ satisfaction with remote work has also emerged as being strongly linked with productivity [41,42].

For instance, employees satisfied with the ICT tools at work are more willing to explore additional features of their systems while also seeking more effective ways to execute their work tasks, thus enhancing their performance and innovativeness [19,43].

In the same line, a longitudinal study carried out in Colombia on employees who compulsorily switched to teleworking because of the pandemic showed that work–home conflict and work overload generated strain, which decreased job satisfaction with telework, and thus perceived job performance [44].

Concerns about contracting the virus have also proven to play a role in productivity: remote work satisfaction in an Italian group of employees was higher for employees with higher perceived productivity and lower concern about the virus [42]. In other words, the relationship between work satisfaction and perceived productivity is moderated by concern about the virus [42]. Not surprisingly, satisfaction with COVID-19 countermeasures among employees working in the office, and thus with employees’ perceived safety, was significantly associated with work productivity [45].

Regarding individual performance, in a study carried out among academic staff in Indonesia, it emerged that digital orientation (i.e., an individual’s commitment towards the application of digital technology to support the accomplishment of the job) impacts employees’ digital capability, which in turn affects their productivity. In other words, digital capability mediates the relationship between digital orientation and productivity [12]. Consistently, a study on software engineers showed low to no suffering due to the home-working condition, thanks to the high familiarity with ICT tools that allowed them to create the best possible conditions at home to pursue their goals [46].

Another factor affecting performance is procrastination, which exerts a highly detrimental impact on individuals’ work effectiveness from home [8]. Indeed procrastination emerged as a problem with productivity at work when not counterbalanced with self-discipline [8,40].

Finally, it emerged also that work-home interference plays a detrimental role in performance. From the interviews collected in the study, it emerged that working parents faced a more significant challenge in balancing work and family duties, especially when working at home and at the same time having to take care of children who were out of school because of the lockdowns. More than that, more working hours were required very often. These interferences between work and family domains could make people feel exhausted and, therefore, less productive [8,40].

### 3.3. Impact of Organizational and Workplace Factors on Individual Performance and Productivity

Generally speaking, the organizational contexts supporting mental, physical, and social functioning have increased employee productivity during the pandemic. In addition, some papers focused on enforcement actions that positively affected employees’ productivity and job satisfaction [37,47,48,49,50,51].

The pandemic induced occupational discomfort, namely the lack of proper telework conditions from home, which impacted job performance. Occupational discomfort refers to the lack of clear policy about working from home, experiencing poor ICT connectivity, inadequate personal space, time management, limited guidance, poor ergonomic premises, and no peer communication [41,48]. Furthermore, all types of multitasking and interruptions from colleagues, supervisors, and their family members, were mentioned as detrimental to employee productivity. It is interesting to note that women are more exposed to these latter problems [52].

Not surprisingly, productivity was enhanced among organizations that provided resources to create a proper work environment at home as well as technical assistance and specific training for the new ways of working [37,48,49,50].

Leadership, of course, plays a relevant role in determining employees’ productivity.

Leaders promoting a sustainable way of working for their employees boosted their organizational commitment and extra-role behavior, which, in turn, are antecedents of higher productivity [5]. Sustainable leadership refers mainly to the ability to offer guidance and avoid intrusive monitoring [8,47]. Positive and effective leadership behavior examples included the ability to acknowledge the quality and quantity of the work done by employees, despite not having the chance to monitor them directly, and, consequently, assigning the right amount of work to prevent workload [7,10,26,36,53,54,55,56,57].

Another valuable leadership skill reported in the papers includes monitoring and promoting social support, especially in sharing digital support among employees [58]. Thanks to these initiatives, employees are helped to overcome reluctance and apprehensiveness related to the use of ICT and their dependency [59]. A further issue tackled by positive and effective leadership leading to productivity is the handling of teams. Introducing new norms and standards about online communication and asynchronous collaboration could overcome conflicts among team members related to delays, interruptions, different individual work rates, or workloads not distributed equally. These problems were a serious menace to team performance and productivity [47].

Finally, communication is another organizational factor linked to performance. A study in China showed that ineffective organizational communication negatively impacted individuals’ work effectiveness from home [8], specifically when employees did not receive the necessary instructions and information to perform their duties.

### 3.4. Impact of the Workplace on Well-Being

Several studies highlight how productivity in the workplace is strongly linked with employees’ physical and mental well-being [39,45,50,60]. The pandemic changed the workers’ routines and lifestyle, generating problems both in physical and mental/psychological health [24].

Regarding physical distress/problems among workers during the lockdowns, several studies report confinement to home and sedentary activity leading to the discomfort of different body parts [61]. Musculoskeletal pain emerged as a consequence of the imposed sedentarism and the inadequacy of the physical premises available at home, such as lack of ergonomic chairs, proper lights, or a private space to work [11]. In addition, new ways of working were introduced in most cases very rapidly, without the opportunity to organize and revise them to prevent distress both at physical and psychological levels [62].

These sudden changes affected working routines and habits. For example, a study in Japan reported that employees who worked remotely instead of in the office because of the pandemic spent less time doing physical exercises compared to colleagues who already worked remotely before the COVID-19 restrictions and those who worked from the office [63]. This finding is in line with the worsening of sleep quality, decrease in work-related health, and decline in physical functioning found in other studies [64]. Consistently, a study in Canada flags screen fatigue problems [65] due to longer work hours while working from home than in the office.

In many cases, the new way of remote work affected the quality of working life due to the difficulties in disconnecting from work, thus working longer hours.

A number of factors related to organizational factors also affected the psychological well-being of employees. For example, a study developed in Romania has shown a positive relationship between remote working and perceived professional development levels, job satisfaction, and well-being [66].

At the same time, several organizational factors affected employees’ well-being, generating depression, anxiety, and stress among employees [19]. These factors include: working extra hours, having a heavier workload, feeling socially isolated, worsening feelings of job security, experiencing difficulties in accessing the necessary work tools from home [67], and feeling a strong demand for new ICT-related skills to cope with the new ways of working. However, it is important to first acknowledge the role of the pandemic itself and the fear of contracting the virus. A study differentiated between the sources of ill-being, showing that a significant part of the anxious and depressive symptoms was due to COVID-19-related conditions instead of work arrangements [37].

Furthermore, factors such as longer working hours and the general home-working arrangement impacted employees’ well-being even in other forms, for example, by compromising work-life balance or reducing the time devoted to leisure, family duties, and friends [68].

The pandemic-related arrangements impacted the quality of work life as well. Isolation made it more challenging to receive and ask for help from colleagues and reduced the quality of interpersonal exchange; isolation also meant problems connected with poor or difficult communication due to the sudden changes in the organization of work [8]. Isolation also undermined the sense of belonging to the organization, which, in turn, caused depressive symptoms [69]. Following this line of thinking, a study carried out among administrative and teaching staff of Iraqi universities found that telework only reduces job stress when employees do not believe it will lead to social isolation [70].

Job demands for new ICT skills and procedures elicited a sense of professional inadequacy among employees, as well as the fear of losing their job and facing financial instability. The latter was also connected to the financial difficulties faced by the organization or the automation of certain job tasks, or the competition with colleagues having higher ICT skills [7]. Several studies refer specifically to techno-stressors, that is, stress factors linked directly to the use of ICT. These include techno-overload, related to the intensification and increased workload connected with the introduction of ICT, and techno-invasion, related to the blurring of boundaries between work and private contexts due to ICT use (see i.e., [16,28,65,71]). Some papers refer to work overload, which is the perception of having too many work-role tasks to fulfill, due to the new way of working and not having enough time to do them, despite the time saved for commuting or going to the office [52]. Linked with it is also reported a required high level of multitasking, as well as work-based interruptions (such as these linked to family tasks), generating lower performance [52].

### 3.5. Impact of Individual Factors on Well-Being

It has to be noted that these factors did not emerge as affecting all workers in the same way. In fact, some groups of workers were more affected than others.

Some studies analyzed the role of gender in the risk for employees’ physical and mental health problems. For example, in a study carried out in India, it emerged that women were more affected by organizational and social stress [72]. Moreover, in a study carried out in Egypt among university staff members, high levels of technostress were linked with the female gender and a lousy workplace environment [73]. Studies comparing women and men show quite a consistent pattern, where women report more stress and more difficulties, worsened by a bad workplace environment [26,52,66,72,73,74,75] as well as a higher number of hours devoted to child care and home tasks [72] in comparison with men. Similarly, a Canadian study showed that marginalized workers (women, migrants, and people facing financial hardships) reported lower job security, which was related to lower well-being scores [76].

Even other demographic factors impacted the quality of work and life during the pandemic. For example, a study carried out in Germany and Switzerland reported that younger age, living alone, reduction of leisure time, and changes in the quantity of time devoted to caring duties were associated with more detrimental psychological outcomes on personal life. On the other hand, living with a partner or family, short-time work, increase in leisure time, and caring duties were associated with positive mental well-being [18].

Social conditions were reported as well. For instance, among a group of school teachers in Chile, more than half suffered from poor mental health, but those more prone to psychological problems were working in private-subsidized schools, working overtime hours [77].

In line with previous literature on stress, a couple of studies addressed the relevance of employees’ activation of proactive coping strategies, such as help-seeking and active problem-focused coping, recreation, and relaxation activities to cope with work-related stress [57,78].

Employees’ cognitive appraisal of their work is also linked to individual well-being: in the case of telework, a study developed in Romania reported a positive relationship between professional development and competencies, job satisfaction, and well-being, and a negative relationship between the emotional dimension, commitment, autonomy, and well-being [66].

Other studies dealt with dispositional traits related to individual well-being. For example, from a study carried out among a group of employees working from home, it emerged that those with a “solitary profile” (i.e., high levels of preference for solitude and neuroticism, low levels of extraversion and agreeableness, and moderate levels of conscientiousness and openness) reported higher loneliness at work, higher levels of stress, and lower levels of job satisfaction and work engagement than those with an “affiliative” profile (i.e., low levels of preference for solitude and neuroticism, high levels of extraversion and agreeableness, and moderate levels of conscientiousness and openness) [79].

Another powerful personal resource affecting individual well-being is self-discipline. Workers who considered themselves self-disciplined claimed to be more able to deal positively with the work-family balance, than those who described themselves as less self-disciplined, completing their work more efficiently and timely [8,80].

Self-compassion also emerged as a competence able to promote employees’ well-being: workers with a higher score in self-compassion show lower levels of depression and loneliness. This association is because self-compassionate employees are more likely to feel connected with others during the challenging experience of work loneliness imposed by the lockdowns. Moreover, they also tend to be more aware of and more ready to accept their feeling of loneliness [69].

In line with this, mindfulness turned out to be positively related to job satisfaction and negatively associated with technostress [19,41]. Although mindful employees are more likely to assess their working conditions, they are more able to respond more objectively to the demands and challenges posed by the new situations, thus lowering the impact of techno stressors and feeling more competent about them their professional skills [19]. 

Similarly, emotional intelligence has also been shown to reduce the negative impact of social isolation on employees’ well-being [81]: in fact, people who hold a higher level of emotional intelligence are more able to perceive, empathize and regulate their emotions, thus becoming more able to develop stronger and more positive relationships with others which, in turn, promotes their well-being [81].

### 3.6. Impact of Organizational Factors on Well-Being

The pandemic has had different effects on employees’ personal lives according to the extent to which it generated dramatic changes and, more than that, on the degree to which employees experienced an opportunity to influence their work and life. How organizations changed and managed employees’ work during the pandemic has proven to impact employees’ well-being [76]. In this line, perceived autonomy and job crafting correlate negatively with stress and positively with workload [82], showing that workers who feel unable to contribute constructively to their job are more prone to psychological distress [83].

Job crafting is crucial when used to grow flexibility and spend more time with family. In these cases, employees show a higher level of psychological health than those who struggle to balance their private life with their working life due to the interference between home and work during remote work and an increase in daily working hours [52]. Consistently, work hours can extend easily when the employees work at home. Hence, leading to an increase in daily working hours [84]. This factor is often combined with another work stressor: role overload. Role overload refers to the extension of the duties and tasks required when the employees work from home, and it was reported to be especially frequent during the pandemic [41,52,67].

Another valuable dimension is job autonomy. Job autonomy, indeed, is negatively correlated to loneliness, suggesting that employees who are, to some extent, free to organize their workday feel a higher sense of connection and relatedness than those who are subjected to different ways of working [8]. On the other hand, in a study carried out in Japan, it emerged that those who were forced to move from office work to telework were less satisfied than those who continued working from home, independently from the outbreak [63].

Job autonomy also refers to employees being able to take care of their mental well-being and alleviate the perception of cognitive overload by implementing “digital detox measures”. Employees applied limits on their use of technologies, for example, switching off notifications, powering off electronic devices at a specific time in the evening, or responding to emails only at a predefined time [85].

Interestingly, autonomy in another study emerged as being correlated negatively with employees’ well-being in the case of telework [66]. Unfortunately, the studies mentioned above do not report on the extent of the autonomy granted to the employees. However, this factor might be related to another factor: the role played by the leadership. The degree of monitoring procedures implemented by leaders, indeed, may undermine a positive work-life balance [86] by impacting employees’ stress and well-being, mainly when performed in an intrusive [8] or authoritative [7] way. Moreover, quality leadership has proven to contrast counterproductive work behaviors and promote organizational citizenship behaviors through internal marketing [51]. At the same time, a lack of “ COVID-19-related informational justice” by the management led to depressive symptoms [69].

On the contrary, positive and effective leadership impacts employees’ well-being [26], contrasting and preventing stress. At the same time, when leaders can promote and sustain organizational and social support, they increase the perception of psychological safety [8,32]. Employees who feel their organization is taking care of them and their work develop positive emotional resources. In these cases, a present and caring leadership style represents a form of adequate organizational support [41].

Moreover, coworkers’ support is linked to a lower workload perception and a more positive work-home balance [8]. Finally, social support elicits emotional resources, making employees more able to cope with the challenges and demands of work [32].

Quality in interpersonal relationships among colleagues and managers has also proven to bring other positive effects. For example, a study carried out in Poland involving 220 IT employees showed that good employee relationships can have a positive effect on job satisfaction. Also, interpersonal trust in managers mediates the relationship between employee relations and job satisfaction [53]. Consistently, another study showed that quality interpersonal relationships with colleagues boost positive coping strategies (i.e., help-seeking and active coping), which promote well-being and quality of work life [57].

In terms of more general management, a study showed the relevance of organizational communication. This factor indeed emerged to be positively associated with employees’ self-efficacy and negatively with technostress and psycho-physical disorders [87]. Furthermore, good organizational communication has also proven to prevent and contrast the feeling of being neglected by the employees, since their commitment to work may be questioned because they are not physically present. Recognizing the work done, in turn, contrasts occupational stress and is linked with employees’ loyalty [88].

## 4. Discussion

This literature review aims to systematize the substantial body of research focusing on the impact of the COVID-19 pandemic on remote working arrangements.

Due to the limitations imposed by the pandemic, workers suddenly switched from working in an office to remote or home-working. Overall, the studies analyzed show heterogeneous consequences on employees’ well-being, productivity and performance. Interestingly, in addition to papers that explored the positive or negative effects of remote working on well-being, productivity or performance [36,39,40,61,63,64,65], other categories of studies have also emerged [42,57,68,78]. These examined how employees’ characteristics shape their remote working experiences, thus influencing their productivity, performance, and well-being. In other words, how individual factors such as personal living conditions (e.g., the management of domestic spaces) [40], personal resources (e.g., coping strategies) [57,78], and perception of ability with ICT [46] influenced their evaluations of remote work. Similarly, organizational factors such as leadership style [5,7,47], co-workers’ support [8], and job autonomy [8,66] have also positively or negatively influenced employees’ perceptions about the transition and implementation of remote work. From this, it can be concluded that remote or home-working is not intrinsically fruitful or harmful, but that personal or organizational factors have characterized its perception. Consequently, during the lockdown periods, remote working represented an opportunity for some employees to increase their quality of life [66,76,83] and a source of personal or work distress for others [8,51,63]. While organizations cannot intervene on personal dispositions towards stress sensitivity, these data are helpful, as they underline which dimensions can be linked to a higher risk while teleworking and which can lead to a higher adaptation and well-being. The organizational factors promoting or mining a fruitful use of remote working can be reorganized and strategically planned to reduce employees’ risks and improve their productivity, performance, or well-being.

An interesting reflection concerns the role of technologies and their perception by employees.

It must be noted that the shift towards remote work through ICT has mainly been addressed during the pandemic as a necessary but also challenging choice for helping firms’ financial and operational sustainability [5]. On the other hand, the impact of a rapid and sudden introduction of ICT in the workplace has often been perceived (and addressed) in relation to the negative consequences of reduced interpersonal interaction among members of the same organization [8]. In this respect, in fact, such an innovation has been often perceived as a serious risk of stress and burnout [19,37]. In reality, the use of technology in most cases was almost the only way to preserve interaction and interpersonal relationships among workers during the lockdowns [27,28]. ICT can also be addressed as a new, different way of interacting among workers, thus presenting not only difficulties but also points of strength [89,90]. The condition under which ICT may jeopardize or promote social support is an aspect that would deserve to be further explored in the future.

On the other hand, a factor that was little explored but could have had a combined effect with work-related stress was the concern for COVID-19 experienced by employees. Fear of getting infected likely made employees feel insecure about their health and safety [91].

Toscano and Zappalà [42] showed a relationship between stress, perceived productivity, remote job satisfaction, and concern for COVID-19. Building on this, it is likely that this aspect influenced findings from other studies not addressing the COVID-19 concerns. Consequently, it would have been helpful to investigate to what extent the pandemic threatened employee productivity and well-being and to verify the specific role of remote work arrangements on the same outcomes. In the other words, it may be useful to distinguish between different factors: the stress generated by the workers’ fear of deteriorating their financial condition in relation to the consequences of the pandemic [7], the concern for their health due to the spread of the COVID-19 [91], the difficulties in dealing with a new way of working (namely, using ICT) [7], as well as the new ways of interacting with other members of the same organization [89].

Future studies on remote working arrangements in different phases of the pandemic management may help figure out how remote conditions themselves impact employees’ experiences of work and its integration with private life.

A further interesting line of study is the role played by interpersonal trust in promoting employees’ well-being and mental health [53,57]). Indeed, as shown in previous studies, interpersonal trust in the workplace has emerged as a strong protective factor after another catastrophic financial event which was the 2008 worldwide financial and economic crisis [92,93,94].

Finally, to the best of our knowledge, only two longitudinal studies [44,95] have been conducted about the effects of remote working on productivity and well-being. Further studies may help to understand the pandemic’s extended consequences and the remote working arrangements, likely allowing to model the specific effects of each condition.

Next to the mentioned detrimental effects, several studies showed how the pandemic led to an unexpected and forced organizational change that created multifaceted conditions of performance, productivity and well-being related to remote working [5]. Even involuntary and unplanned changes imply organizational structure or process transformations [96]. They can be addressed through a cycle of structured actions, including establishing a sense of urgency, forming a powerful leading coalition, creating and communicating a vision, enabling others to act on the vision, planning and creating short-term victories, consolidating improvements to bringing about more change, and institutionalizing new approaches [97]. These models highlight that a deep understanding of employee attitudes and behaviors towards such changes is essential to address them successfully and minimize negative consequences [98]. On the contrary, if this condition is not satisfied, employees may show adverse reactions, such as resistance, resentment, and disengagement, that can inhibit the successful implementation of organizational change [99].

A general consideration must be made about the type of remote working conditions studied during the pandemic. COVID-19-related remote working conditions, indeed, required a peculiar form of adaptation and adjustment for several reasons [48]. Firstly, remote working before the pandemic represented an alternative to office work offered to employees to promote a higher work-life integration [100]. During the pandemic, instead, it became a full-time mandatory practice, thus losing the voluntary nature that characterized it [48]. Secondly, remote working was considered the only tool to ensure corporate continuity during the crisis. However, several organizations were not ready (from a strategic, instrumental, and technological point of view) to implement this new arrangement [6]. In this regard, the organizations that showed valuable abilities to manage the change process related to the pandemic were even able to reduce the impact of potential stressors on the employees by driving them in the change and implementing practical actions to support their well-being, performance, and productivity [49,50].

## 5. Conclusions

It is unknown what will be left of remote work and in which organizational contexts it will persist since many national contexts are still elaborating laws and regulations to define the administrative boundaries of such practices [101,102].

Indeed, the COVID-19 pandemic and the following organizational changes brought positive consequences. Organizations, especially leaders, confirmed that many good practices concerning increasing ad hoc skills in managing remote work and strategies and positive habits promoting psychological and physical well-being could be implemented. In this regard, the review’s collected studies have shown a wide range of mental and physical health promotion programs, new approaches to online training, and creative ways of socializing at a distance. These strategies have been shown to effectively alleviate potential work stressors and improve the workers’ adjustment to remote work [27,37,49,50,103]. At the same time, virtual learning and development, communication exercises, live sessions for training new skills, digital classroom training modules, e-learning modules, and many other creative learning sessions have been the starting point to sustain teleworkers’ performance during the COVID-19 pandemic [49]. Moreover, such programs were often paired with technical and ergonomic resource provision, even through technical assistance and specific trainings [37,48,49,50]. These practical forms of support boosted other forms of organizational communications around the vision of change and rewarding procedures involving employees [51]. Overall, the studies showed that organizations that provided both instrumental and relational support have effectively managed organizational change and allowed the employees to adjust to remote work.

The enforcement actions developed during the pandemic will be described in more detail in the following section dealing with managerial implications. In fact, they can also represent useful intervention methods in the future context of hybrid work.

### 5.1. Managerial Implications

Organizational factors that promote or hinder an effective use of remote working can be strategically reorganized and planned to improve employees’ productivity and well-being. Our results highlight which dimensions can be considered obstacles in the remote or home-working practice and which ones can lead to a greater adaptation and well-being.

Several studies showed how some organizations endorsed positive actions with COVID-19-related work issues and supported employees’ well-being and productivity.

During the pandemic, organizations supported employees’ social well-being by implementing engagement actions and promoting a culture of trust and collaboration, thus promoting their social well-being [37,47,49,50,53].

Most of these programs have focused on promoting employees’ mental and social well-being [27,37,49,50,53,103].

Some organizations have introduced webinars focusing on anxiety and stress, online meditation classes, and training to develop new daily habits about health, hygiene, and the positive work-life balance of employees [49]. Other organizations have also provided access to yoga and fitness instructors to compensate for sedentary lifestyles and lack of physical activity in teleworkers, which, together with harmful eating habits, can lead to severe issues (such as obesity, cardiovascular disease, diabetes mellitus) [50].

Some activities aimed to empower workers, increase awareness of the current situation, and analyze the pros and cons of remote/home-working [37,49]. In the academic context, the researchers were involved in virtual empowering sessions on different topics, such as individual differences in addressing the COVID-19 challenges, managing the crisis, and how to thrive during and not just survive [37].

Other developed actions to motivate the teams have been weekly alignment sessions, team meet-ups, virtual challenges and competitions, online courses, sharing content such as TED Talks or online books, webinars with industry experts, and online counseling sessions. Organizational support was also expressed through “family engagement practices” as intended online babysitting, in which employee kids were kept engaged for a few hours while parents worked from home [49]. At the same time, other practices had a more informal and playful character [50]. For example, in some organizations, the leaders organized team-building activities and virtual events such as lunch in a video conference [49], online happy hour, hidden talent show, virtual karaoke, and campfire challenges [50].

Each of these actions helped develop a positive relationship between superiors and employees so that these last members felt free to discuss any issues and concerns [50].

More frequently, team leaders had more individual online meetings with other members to check their mental health and assess the team’s mood [53]. Teams have also independently developed peer support actions [21,47].

Employees established fifteen-minute “online morning huddles”. The purpose of these informal meetings was to take care of others and check their well-being through questions that concerned not the work but the personal sphere of colleagues (e.g., family). In addition to stemming the sense of loneliness, this good practice has helped develop a compassionate culture among members and created cohesion [21].

These listed are examples of good practices that have had a positive impact on employees working outside the workplace during the pandemic. These executive actions, developed in a pandemic context, could continue to be used even in the context of normal life or still characterized by restrictions representing precious approaches to intervention for promoting employees’ productivity and well-being in a sort of “heterogeneity of purposes” [104].

### 5.2. Practical Implications

A section of this paper deals with effective ways and interventions to address stress and challenges posed by the COVID-19 pandemic, in the hope that such information will constitute a resource for managers and HR consultants.

### 5.3. Challenges, Risks and Opportunities

The main challenge will be to adapt these new ways of working in a post-pandemic situation. This process could include various factors, such as the type of organization, the size of the organization, the organizational culture, and the implicit norms already present within each organizational reality. Furthermore, leaders could model the perception of ICT use, helping employees to perceive technology as a means of facilitating the performance of tasks and professional and social interactions.

However, as highlighted by some studies in this review, the main risk is to organize flexible work arrangements forgetting their initial assumptions instead of increasing the employees’ autonomy and flexibility and improving their work-life integration.

Regarding the opportunities, organizations could further apply the enforcement actions developed during the pandemic. These good practices can have positive effects on employees’ well-being and productivity also in a post-pandemic work environment.

Furthermore, the consistent post-pandemic use of flexible work arrangements can promote research on the impact of technology-mediated strategies promoting employees’ flexibility and autonomy on their well-being and productivity. In this regard, longitudinal research designs could be used to have a better picture of the phenomenon.

### 5.4. Limitations and Strengths

Our study took into consideration peer-reviewed papers, published in English in international journals. We realized, though, that there is much more material published in other languages, which might have offered interesting insights. Moreover, other papers about the pandemic’s consequences on the workplace and employees’ well-being may be published soon. On the other hand, it was important to start a reflection based on the studies already carried out and deepen the knowledge about this topic.

This is exactly the main strength of this paper. Having a detailed view of workplace changes and valuable indicators such as employees’ performance, productivity and well-being can enable future work organization development and how to deal with new challenges.
